# The overexpression of long intergenic ncRNA00162 induced by RelA/p65 promotes growth of pancreatic ductal adenocarcinoma

**DOI:** 10.1111/cpr.12805

**Published:** 2020-05-04

**Authors:** Yu Lu, Min Wu, Jie Fu, Yichen Sun, Kenei Furukawa, Jianhua Ling, Xue Qin, Paul J. Chiao

**Affiliations:** ^1^ Department of Clinical Laboratory Liuzhou People's Hospital Liuzhou China; ^2^ Department of Molecular & Cellular Oncology The University of Texas MD Anderson Cancer Center Houston TX USA; ^3^ Department of Clinical Laboratory First Affiliated Hospital of Guangxi Medical University Nanning China

**Keywords:** LINC00162, lncRNA, Pancreatic ductal adenocarcinoma, RelA/p65, RNA sequence

## Abstract

**Objectives:**

Recent observations have emphasized the role of long non‐coding RNA (lncRNA) in cancer progression; however, a genetic profile of lncRNAs in pancreatic ductal adenocarcinoma (PDAC) remains an ongoing study.

**Materials and Methods:**

In this research, RNA sequencing showed that LINC00162 is dramatically increased in patient‐derived tumour cell lines (PATC) compared with the human pancreatic nestin‐positive epithelial (HPNE) cells.

**Results:**

These data were validated in several PDAC cell lines, and significant upregulation of LINC00162 was found in all of them. Knock‐down of LINC00162 significantly inhibited the proliferation, colony formation and migration of PATC cells in vitro and suppressed the growth of PATC xenografts in vivo. Overexpression of LINC00162 in PDAC cell lines (AsPc‐1) showed consistent results, with significantly increased proliferation, colony formation and migration of AsPc‐1 cells, as well as enhanced tumour growth of the AsPc‐1 xenografts in vivo. Furthermore, the result of Chromatin immunoprecipitation assay revealed that RelA/p65 directly bound to LINC00162, and the expression of LINC00162 in PDAC decreased after RelA/p65 knock‐down, the proliferation ability of AsPc‐1 also significantly inhibited after knocking down LINC00162 and RelA/p65 simultaneously, indicating that RelA/p65 directly involve in the transcriptional regulation of LINC00162.

**Conclusions:**

In sum, our results provide first evidence for the role of LINC00162 in promoting PDAC progression and the potential underlying mechanism of LINC00162 overexpression.

## 1 INTRODUCTION

Pancreatic ductal adenocarcinoma (PDAC), with 53 070 new cases in 2016, is the fourth leading cause of cancer mortality in the United States.^1^ Its 5‐year overall survival rate has remained at <10%, mainly due to difficulties in early diagnosis, its easy metastasis, and its unresponsiveness to most treatment. Hence, examining the molecular biological process underlying PDAC and identifying novel detection biomarkers will help us better understand this disease and develop effective targeted therapies.

Advances in large scale genome analyses have identified several most commonly detected mutations and alterations as the signatures of PDAC,^2^ such as KRAS mutation‐an early event in PDAC development that has been detected in 80%‐95% of PDAC; inactivation of *Ink4a* and *p53* tumour‐suppressor genes, identified by Hruban in approximately 50%‐75% of PDAC cases; and the loss of CYLD, identified by Vogelstein's lab in a comprehensive genetic analysis of 24 PDACs.^3^ Among all these findings, most of the researchers focus on the protein‐coding genes. However, according to the genome data, sequences of protein‐coding genes comprise <3% of the human genome, while the majority of the remaining genomic sequences are transcribed into non‐coding RNAs, indicating that the human transcriptome consists largely of non‐coding RNAs.^4^

Recently, long non‐coding RNAs (lncRNAs), which exceed 200 nucleotides in length, have attracted more attention in cancer research and increasing evidence has suggested that lncRNAs constitute an important component of tumour biology. For instance, by reprogramming the chromatin state, the overexpression of the lncRNA HOTAIR was found to promote the invasiveness and metastasis of breast cancer cells, and its expression level in primary tumours was determined to be a potent predictor of final metastasis and death in breast cancer.^5^ The lncRNA PCAT19 also interacts with HNRNPAB to activate a subset of cell‐cycle genes associated with prostate cancer progression, thus promoting prostate cancer growth and metastasis.^6^ Guo et al^7^ found that lncRNA SNHG6 is not only an oncogene in hepatocarcinogenesis but also a potential prognostic indicator for hepatocellular carcinoma (HCC), and, by inhibiting S‐adenosylmethionine production in HCC, dysregulation of SNHG6 can lead to aberrant genome‐wide hypomethylation and further disturb the treatment of HCC. Therefore, lncRNAs are pervasively transcribed and contribute to the hallmarks of various cancers.^4^ However, to the authors’ knowledge, few studies consider lncRNAs expression profiles in PDAC, and the potential clinicopathological significance of lncRNAs in PDAC remains unclear.

In the present study, a genetic profile of lncRNA in PDAC was carried out. The researchers screened gene expression profiles in four cells established from patient‐derived xenografts of PDAC, as well as hTERT‐immortalized human pancreatic epithelial nestin‐expressing cells (HPNE) by RNA sequencing. Using this data, the expression of selected lncRNAs was further validated and integrated functional studies were conducted. This study aimed at providing an in‐depth understanding of lncRNA in carcinogenesis and identifying clinically relevant targets for PDAC.

## 2 MATERIALS AND METHODS

### 2.1 Cell lines and culture

The human pancreatic cancer cell lines (AsPc‐1, BxPc‐3, Panc‐1, Pan‐28 and Miapaca 2) were purchased from the American Type Culture Collection (Manassas, Virginia, USA) and cultured under conditions specified by the supplier. PATC‐43, PATC‐50, PATC‐53 and PATC‐66 were established from patient‐derived xenografts provided by Dr Jason B. Fleming (MD Anderson Cancer Center, Texas, USA).^8^ The hTERT‐immortalized human pancreatic epithelial nestin‐expressing (HPNE) cells and the immortalized/non‐tumorigenic HPDE cells were described elsewhere by previous research.^9^, ^10^ Other cell lines, including Panc‐1/shRelA/p65 (shRNA1 and 3) and AsPc‐1/shRelA/p65 (shRNA1 and 3) ‐ were established in Dr Chiao's laboratory and were cultured as previously described.^9^, ^11^

### 2.2 RNA sequencing study and quantitative reverse transcription‐PCR

Total RNA was isolated from PATC‐43, PATC‐50, PATC‐53 and PATC‐66 and from control cells (HPNE) using Trizol (Life Technologies) according to the manufacturer's protocol. RNA sequencing was performed on the Ion Proton platform at the MD Anderson Cancer Center Core Facility. The comparison of the lncRNA expression profiles among these groups was performed using the tophat2 and cuffdiff standard procedure. The lncRNAs with at least 2.0‐fold changes and *P* < .05 between the study and control groups were considered significantly different expressions.

Quantitative real‐time PCR (qRT‐PCR) was performed for LINC00162 in different cells. Total RNA was isolated and converted to cDNA by reverse transcription using an iScript cDNA Synthesis Kit (Bio‐Rad). The qRT‐PCR reactions were carried out with SYBR reagent using an IQ5 Multicolour real‐time PCR Detection System (Bio‐Rad), and data were normalized to β‐actin. Primers of these genes were described as follows: β‐actin, forward primer 5′‐catgtacgttgctatccaggc‐3′, reverse primer 5′‐ctccttaatgtcacgcacgat‐3′; LINC00162, forward primer 5′‐cctccagcccagtccttt‐3′, reverse primer 5′‐cacatgtgctccccacct‐3′.

### 2.3 Fluorescence in situ hybridization (FISH)

LncRNA expression was evaluated by FISH using Stellaris^®^ RNA FISH Probes and Methods (LGC Biosearch Technologies). Custom Stellaris^®^ FISH Probes were designed against LINC00162 (NC_000021.9) by utilizing the Stellaris^®^ RNA FISH Probe Designer (Biosearch Technologies, Inc) available online at http://www.biosearchtech.com/stellarisdesigner. The PDAC cells were hybridized with the LINC00162 Stellaris^®^ RNA FISH Probe set labelled with Quasar^®^ 570 Dye (Biosearch Technologies, Inc), following the manufacturer's instructions available online at http://www.biosearchtech.com/stellarisprotocols. Briefly, PDAC cells were grown on 18 mm round coverglass in a 12‐well cell culture plate. They were fixed with fixation buffer for ten minutes and permeabilized in 70% ethanol for 1 hour before being hybridized overnight with a 125 nmol/L LINC00162 specific probe set at 37°C. 4’,6‐diamidino‐2‐phenylindole (DAPI; Sigma‐Aldrich) was used for nuclear staining. The fluorescence signals of LINC00162 and DAPI were observed using Zeiss LSM 710 (Carl Zeiss).

### 2.4 Lentiviral transfection

Human lentiviral shRNA against human LINC00162 was designed. The sequence for LINC00162 was 5′‐GCTCTATGACCTCATCCAT‐3′ (shLINC00162‐1) and 5′‐GGACTTTCAAGAGGTAAAT‐3′ (shLINC00162‐2). The shRNA oligos were purified, reannealed and then cloned into lentiviral vector pLV‐H1‐EF1a‐red (Cat#SORT‐B11, Bioserttia Inc). Lentiviruses were generated by transfecting the above vectors into 293T cells. After 48 hours transfection, viral culture supernatants from 293T cells were collected, filtered through a 0.45‐µm filter, and used to infect the 50% confluence PATC‐43 and PATC‐50 cells in the presence of 8µg/mL polybrene (Sigma‐Aldrich Corporation). For control purposes, cells were infected with Lentivirus carrying scramble shRNA. After 48‐72 hours of infection, cells were passaged, and drug selection of infected cells was performed with puromycin (Invitrogen Life Technologies, Inc) to purify polyclonal‐infected populations of LINC00162‐shRNA‐expressing cells. The effectiveness of shRNA knock‐down was confirmed by qRT‐PCR and FISH.

AsPc‐1 was used to establish LINC00162 high‐level expression cell lines, where it was transfected by the lentivirus expressing LINC00162 and GFP with puromycin resistance. The following lentivirus production, infection procedures, and drug selections were performed as described above for the lentivirus.

### 2.5 Cell proliferation and colony formation

PDAC cells infected with lentiviral vector were seeded in 100uL medium into a 96‐well plate for 24, 48, 72, 96 and 120 hours, respectively. At the indicated time points, cells were treated with 10 μL 2‐(4‐indophenyl)‐3‐(4‐nitrophenyl)‐5‐(2,4‐disulphophenyl)‐2H‐tetrazolium monosodium salt reagent (CCK8, Dojindo Laboratories) and incubated for 1 hour. Then, the absorbance of each well was measured on a plate reader at 450 nm.

To evaluate the monolayer colony formation of stably transfected PDAC cells, the cells were plated into 6‐well plates at a density of 200 cells per well and incubated for 14 days. Then cells were fixed with 4% paraformaldehyde and stained with 1% crystal violet for 15 minutes. The number of clones (≥50 cells) was observed and counted manually under light microscopy. Each experiment was performed in triplicate.

### 2.6 Cell migration assay

Cell migration assay was performed using a Transwell chamber with a polycarbonate membrane containing 8.0‐μm pores (BD Biosciences). Cells were resuspended in serum‐free medium and plated in the upper chamber at a concentration of 5 × 10^4^ cells/well. The upper chamber was then inserted into a well of a 24‐well plate containing 600mL serum‐containing medium (20%). After incubation for 24 hours, the non‐migrating cells in the upper chamber were wiped with cotton swabs and removed, and those that had migrated through the pores were fixed with 4% paraformaldehyde for 15 minutes and stained with 1% crystal violet for 15 minutes. The migrated cells in each chamber were observed at 100× magnification; three random fields were chosen to count and determine the mean number for each group.

### 2.7 Animal model

The orthotopic pancreatic tumour‐bearing mouse model was established as described in previous research.^12^, ^13^ Briefly, female NOD/SCID mice, aged 5‐6 weeks, were purchased from Charles River Laboratories International. When transfected PDAC cell confluence reached 80%, they were harvested, washed twice with PBS and resuspended in PBS buffer with 10% matrigel at a concentration of 1 × 10^6^ cells/50 µL. For orthotopic implantation, 1 × 10^6^ tumour cells were injected subcapsularly in a region of the pancreas just beneath the spleen. The mice were sacrificed eight weeks after injection. Tumours were excised, weighed, embedded in paraffin and stained with haematoxylin and eosin (H&E). Proliferating cells were identified by immunohistochemistry (IHC) for Ki‐67 using rabbit anti‐Ki67 antibody (RM‐9106‐s, Thermo Fisher Scientific). Standard procedures were carried out according to the manual previously described,^14^ and the relative number of Ki‐67‐positive cells was determined by counting nuclear staining cells at 20× magnification in all tumour sections with Image J Software. The experiments with mouse xenografts was approved by the Institutional Animal Care and Use Committee (IACUC) of MD Anderson Cancer Center.

### 2.8 Chromatin immunoprecipitation (ChIP) assay

AsPc‐1 cells were fixed with 1% formaldehyde for 10 minutes for the cross‐link between protein and DNA, followed by quenching with 0.125 M glycine for 5 minutes. Then, DNA was sonicated into fragmented and enriched with anti‐p65(#8242, CST) ( anti‐p65 enriched group), Histone H3 (D2B12) XP^®^ Rabbit mAb (#4620, CST) (positive control) and Normal Rabbit IgG Antibody (#2729, CST) (negative control) at 4°C overnight, followed by incubation with ChIP‐Grade Protein G Agarose Beads (#9006, CST) at 4°C for 2 hours, and the complex was washed and eluted by elution buffer. Lastly, the enriched fragments were subjected to PCR and further analysed by 2% agarose gel electrophoresis.

### 2.9 Assessment of LINC00162 Expression in ‐ RelA/p65 knock‐down cells and rescues experiment

RelA/p65 knock‐down cells AsPc‐1 were established as mentioned above, and the expression of LINC00162 in these cells was detected using qRT‐PCR and FISH. A rescue experiment was also performed by knocking down LINC00162 and RelA/p65 simultaneously to verify the regulation between RelA/p65 and LINC00162.

### 2.10 Statistical analysis

For error bars in all experiments, the standard deviation (sd) was calculated from three independent experiments, and the data are expressed as mean ± sd. For comparison of the significant differences among more than two groups, one‐way ANOVA and Newman Keul's multiple comparison tests were used, and other differences were evaluated by the Student unpaired *t*‐test. All statistical analyses were performed using a commercially available statistical software package (SPSS for Windows, 16.0), and differences reached statistical significance with *P* < .05.

## 3 RESULTS

### 3.1 Expression of LINC00162 is dramatically upregulated in PATC and PDAC cells

**FIGURE 1:**
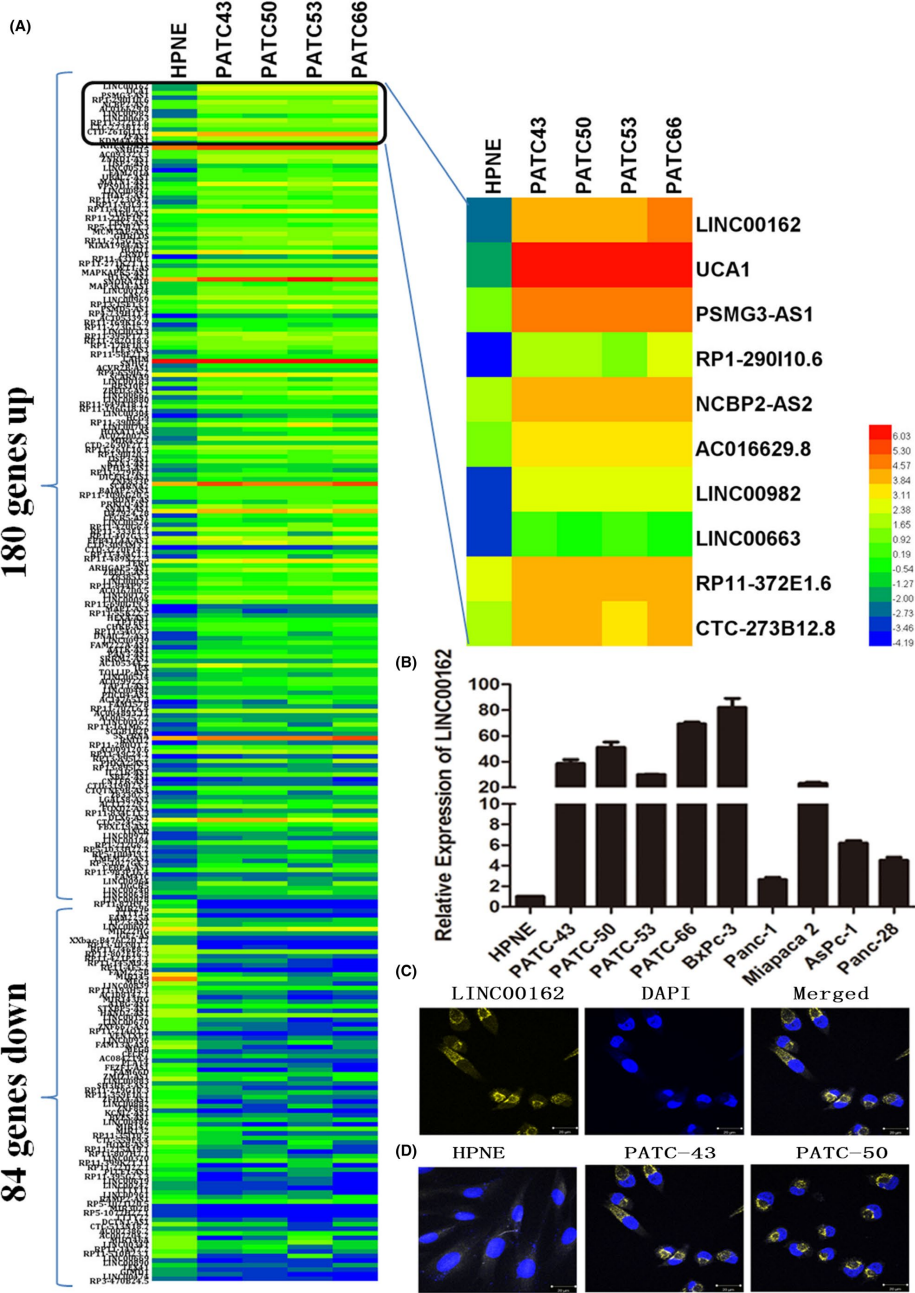
Expression of LINC00162 is specially increased in PATC and PDAC cells. A, The heat map of an RNA sequencing study showed significantly upregulated lncRNA in PATC cells when compared with HPNE, with LINC00162 being the most upregulated; (B) The differential expression of LINC00162 was further validated in a series of PATC and PDAC cells, and an upregulation of LINC00162 was found in all of them, compared with HPNE; (C) FISH results showed that LINC00162 is mainly located in cytoplasm; (D) Higher signals of LINC00162 were observed in PATC cells, but not in HPNE cells, by RNA FISH

HPNE cells are immortalized human pancreatic epithelial nestin‐expressing cells, which have properties similar to that of the intermediary cells produced during acinar‐to‐ductal metaplasia. In this study, PATC cells (PATC‐43, PATC‐50, PATC‐53 and PATC‐66) were established from patient‐derived xenografts. A genetic profile of lncRNA in PDAC was thus carried out in PATC cells, and HPNE cells were utilized as a control to investigate the differentially expressed lncRNA in PDAC. As showed in Figure 1, 264 lncRNAs were found differentially expressed in PATC cells when compared with HPNE; among them, 180 lncRNAs were significantly upregulated in PATC cells, with 84 significantly downregulated, and LINC00162 was the most upregulated lncRNA in all PATC cells (average fold change = 129.07, *P* value = 1.8 × 10E‐8) (Figure 1A). The differential expression of LINC00162 was further confirmed in a series of PATC (PATC‐43, PATC‐50, PATC‐53 and PATC‐66) and PDAC cells (AsPc‐1, BxPc‐3, Panc‐1, Miapaca 2, Panc‐28) by qRT‐PCR, and an upregulation of LINC00162 was found in all of them, compared with HPNE (Figure 1B). To detect the location of LINC00162, RNA FISH was used. The results showed that LINC00162 was mainly located in cytoplasm (Figure 1C), and higher signal was observed in PATC cells but not in HPNE cells (Figure 1D).

### 3.2 LINC00162 affects proliferation, colony formation and migration of PATC cells

**FIGURE 2:**
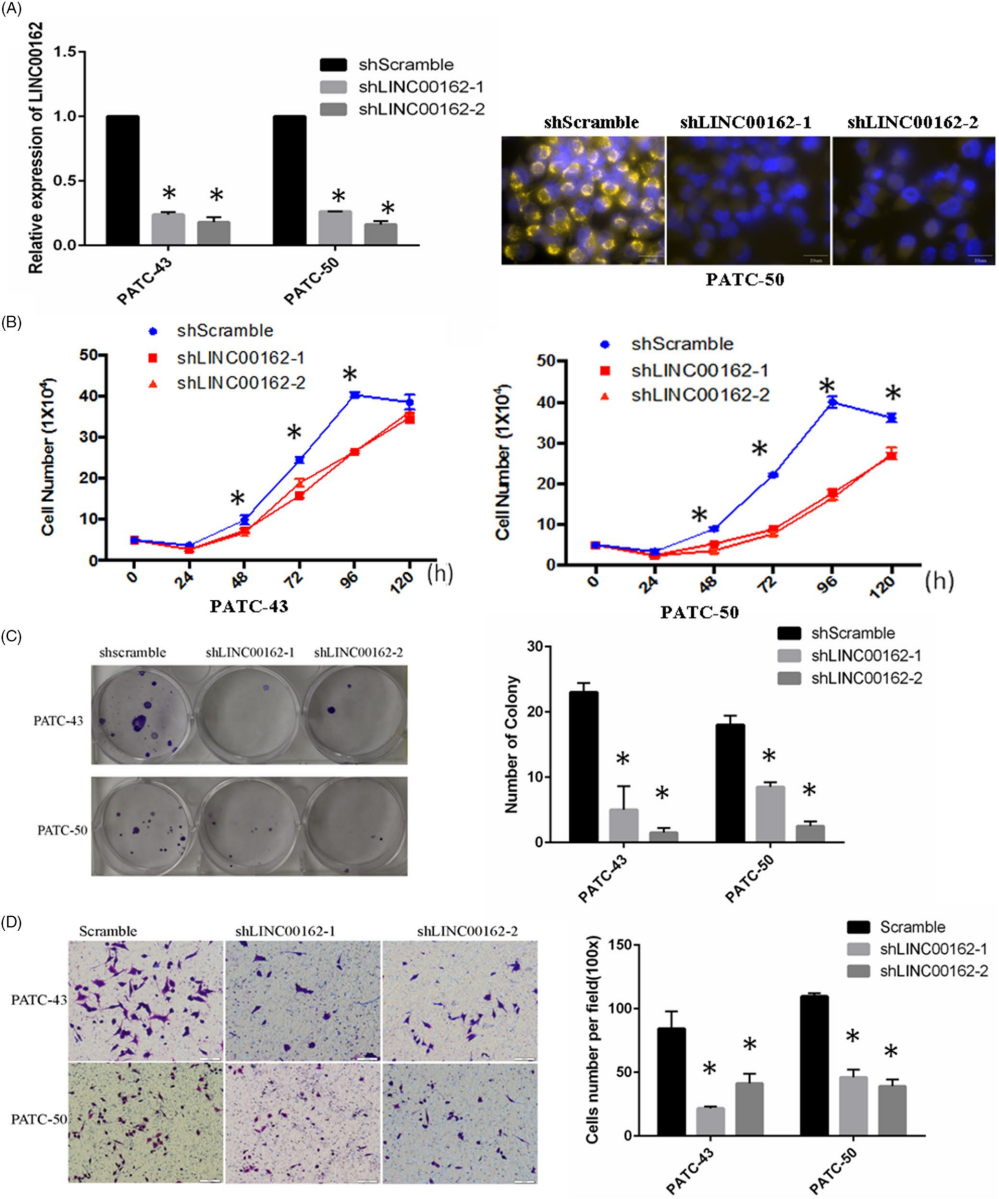
Knock‐down of LINC00162 significantly inhibited proliferation, colony formation and migration of PATC cells. A, The knock‐down effect of LINC00162 in PATC‐43 and PATC‐50 cells was validated using qRT‐PCR and RNA‐FISH; (B) Proliferation assay showed that the number of viable PATC‐43 and PATC‐50 cells was significantly decreased after LINC00162 knock‐down; (C) The colony formation assay revealed that inhibition of LINC00162 substantially impaired cell colony formation ability; (D) Migration assays revealed that knock‐down of LINC00162 decreased cell migration significantly in comparison with scramble cells

To study the cellular functions of LINC00162, two PATC cell lines and one PDAC cell line were selected to carry out loss‐of‐function and gain‐of‐function experiments, respectively. PATC‐43 and PATC‐50 cells were transfected with two LINC00162 targeted shRNAs and a control scramble shRNA. Effective knock‐down of LINC00162 expression in PATC cells was detected using qRT‐PCR and RNA FISH. Analysis with qRT‐PCR indicated that LINC00162 expression was decreased by 74%‐76% with shRNA1 and by 82%‐84% with shRNA2, as compared with scramble shRNA. A similar result was also found in RNA‐FISH. The signal of LINC00162 in PATC‐50 became much weaker after being transfected with shRNA1 and shRNA2 (Figure 2A). Analysis of cell proliferation revealed that the number of viable PATC‐43 and PATC‐50 cells was significantly decreased after LINC00162 knock‐down compared with scramble cells (Figure 2B). The colony formation assay revealed that inhibition of LINC00162 substantially impaired cell colony formation ability, as indicated by the formation of fewer and smaller colonies in PATC‐43 and PATC‐50 cells (Figure 2C). In addition, migration assays revealed that knock‐down of LINC00162 decreased cell migration significantly in comparison with scramble cells (Figure 2D).

**FIGURE 3:**
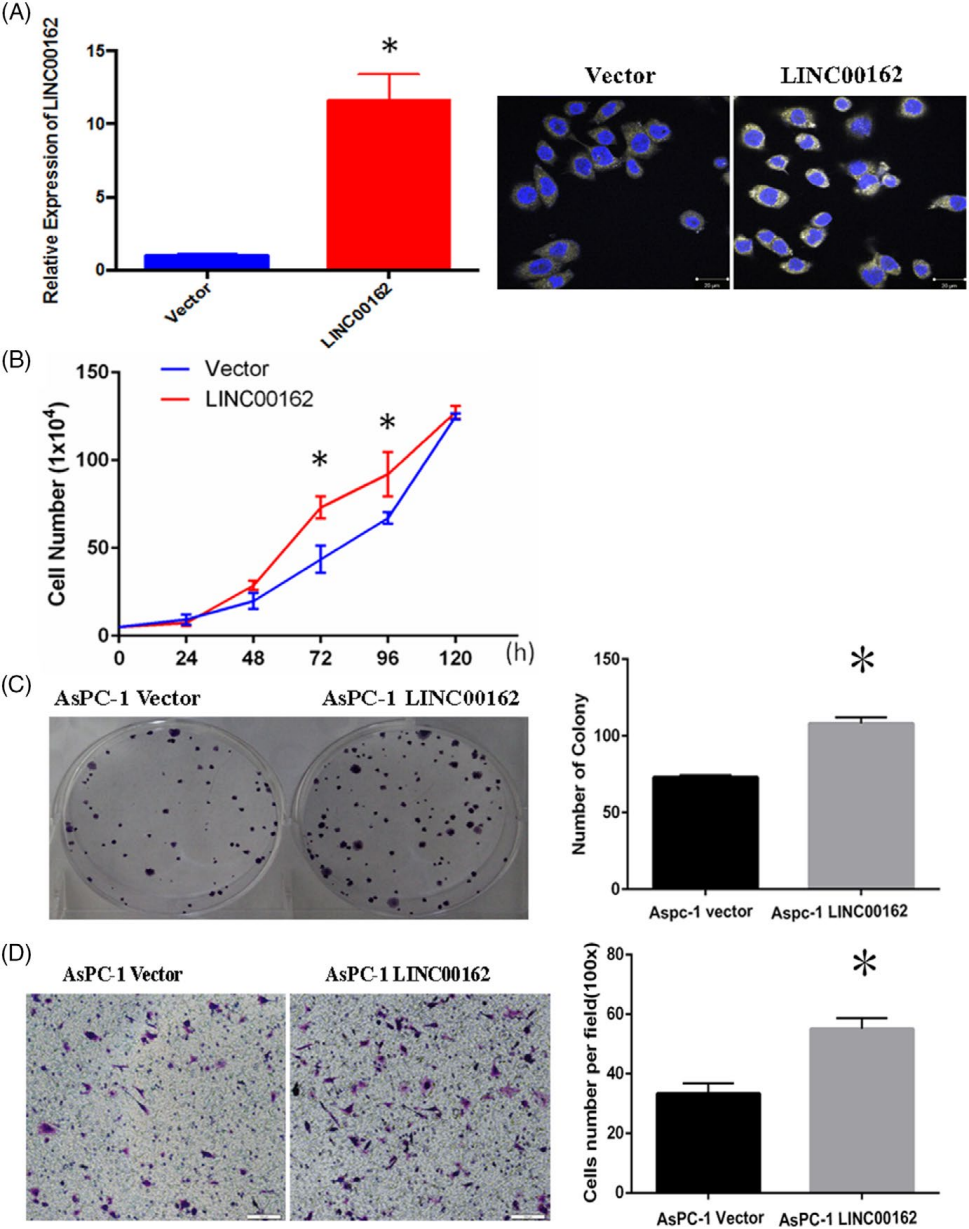
Overexpression of LINC00162 in AsPc‐1 (A) significantly promoted the proliferation (B), colony formation (C) and migration (D) of PATC cells

To further confirm these findings, several gain‐of‐function studies were conducted by overexpression of LINC00162 in one of the pancreatic cancer cells, AsPc‐1. The overexpression effect was also confirmed by qRT‐PCR and RNA FISH (Figure 3A), and consistent results were observed, with a significantly increased proliferation, colony formation and migration ability of AsPc‐1 cells after LINC00162 overexpression, compared with the control vector (Figure 3B‐D). These data provide evidence of the growth promoting role of LINC00162 in PDAC.

### 3.3 LINC00162 affects growth of PDAC xenografts in vivo

**FIGURE 4:**
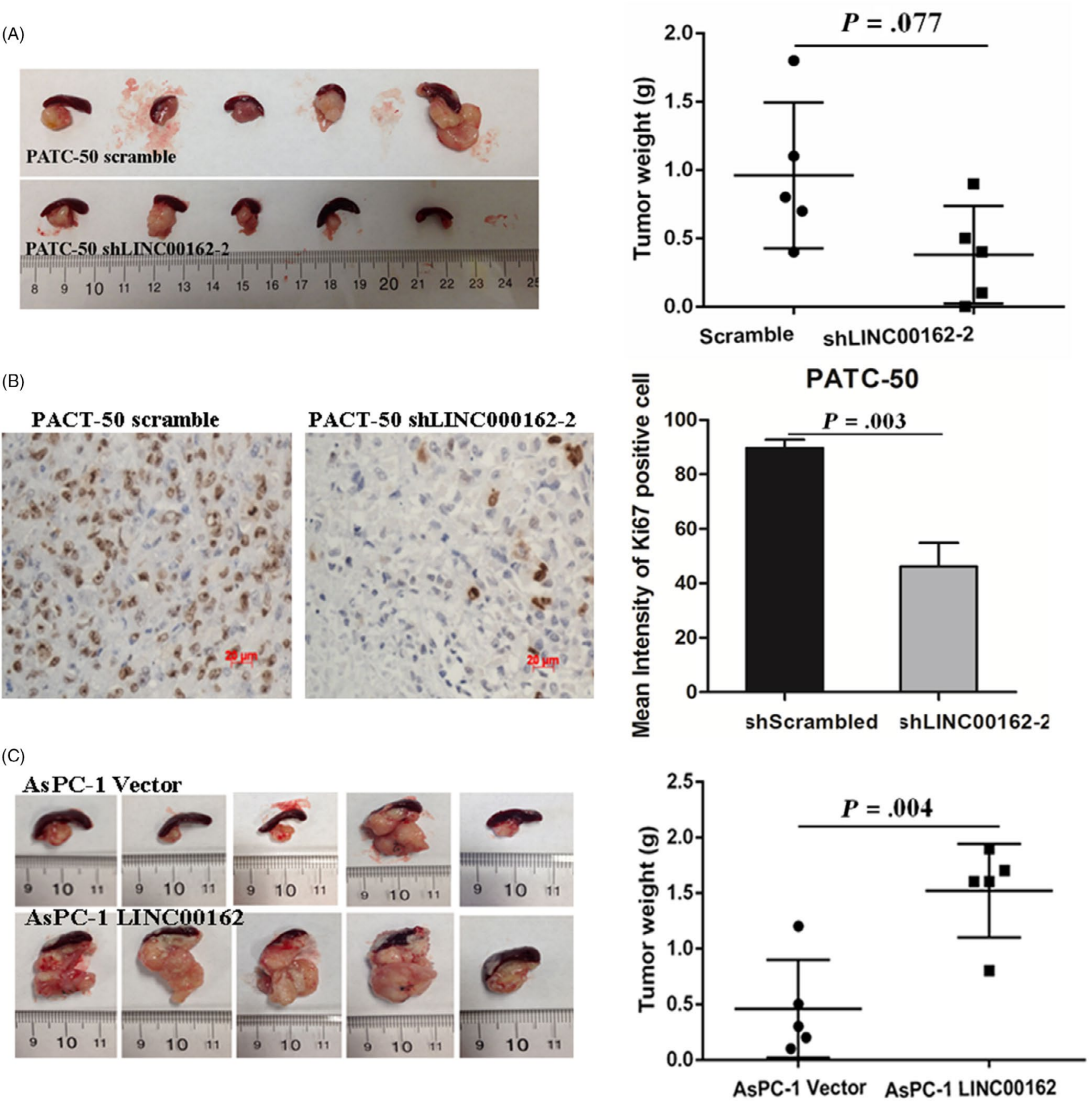
LINC00162 affects growth of PDAC xenografts in vivo. A, Knock‐down of LINC00162 in PATC‐50 resulted in a inhibition of the tumour growth compared with the control tumours; (B) The relative number of Ki‐67 positive cells was decreased after LINC00162 knock‐down; (C) Overexpression of LINC00162 significantly promoted PDAC tumour growth in AsPc‐1

The role of LINC00162 in PDAC progression in vivo was investigated using a PDAC xenograft model in the NOD/SCID mice. PATC‐50 cells, which were transfected with LINC00162 shRNA2 and scramble shRNA, were injected subcapsularly in a region of the pancreas just beneath the spleen. The mice were sacrificed, and the tumour growth was analysed, eight weeks after injection. Knock‐down of LINC00162 resulted in inhibition of the tumour growth compared with the control tumours, though this did not reach statistical significance, perhaps due to the relatively small number of included mice (Figure 4A). When considering the relative number of proliferating (Ki‐67 positive) tumour cells, a significantly lower number of Ki‐67 positive cells was found in xenografts established with PATC‐50 cells transfected with LINC00162 shRNA2, as compared with the control shRNA tumours (Figure 4B). Furthermore, when AsPc‐1 cells with of overexpression LINC00162 and the control vector were injected, the mass of the LINC00162 overexpression tumour was significantly higher compared to the control tumours (Figure 4C).

### 3.4 RelA/p65 directly involves in the transcriptional regulation of LINC00162

**FIGURE 5:**
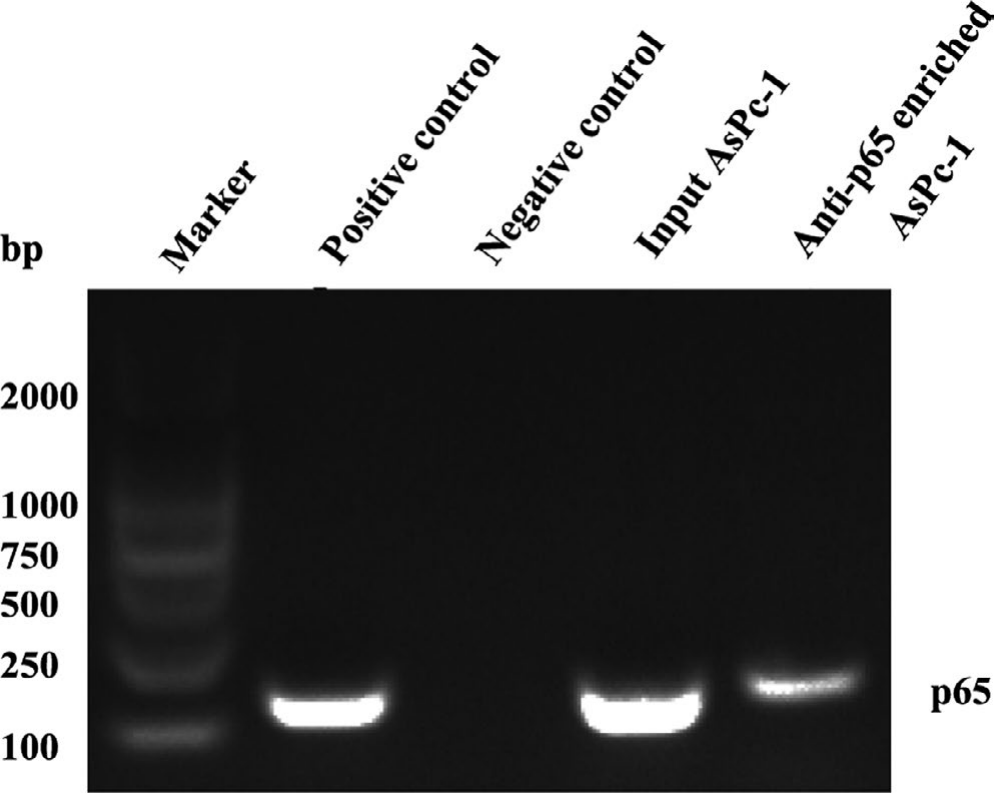
RelA/p65 directly bound to LINC00162. ChIP assay in AsPc‐1 cells using p65 antibody, Histone H3 (D2B12) XP^®^ Rabbit mAb and Normal Rabbit IgG Antibody, followed by PCR and agarose gel electrophoresis

To investigate which transcription factors (TF) may be involved in the transcriptional regulation of LINC00162 in PDAC, animal transcription factor database (http://bioinfo.life.hust.edu.cn/AnimalTFDB) was used to analyse the TF binding site of LINC00162. This analysis found a cluster of TF binding sites in this region, including FOXA1, E2F7, RELA etc, a ChIP assay focusing on RelA/p65 was then carried out to verify this prediction. As showed in Figure 5, positive lanes were found in the input group, positive control group and anti‐p65 enriched group, while no lane was observed in the negative control group, indicating that RelA/p65 directly bound to LINC00162.

**FIGURE 6:**
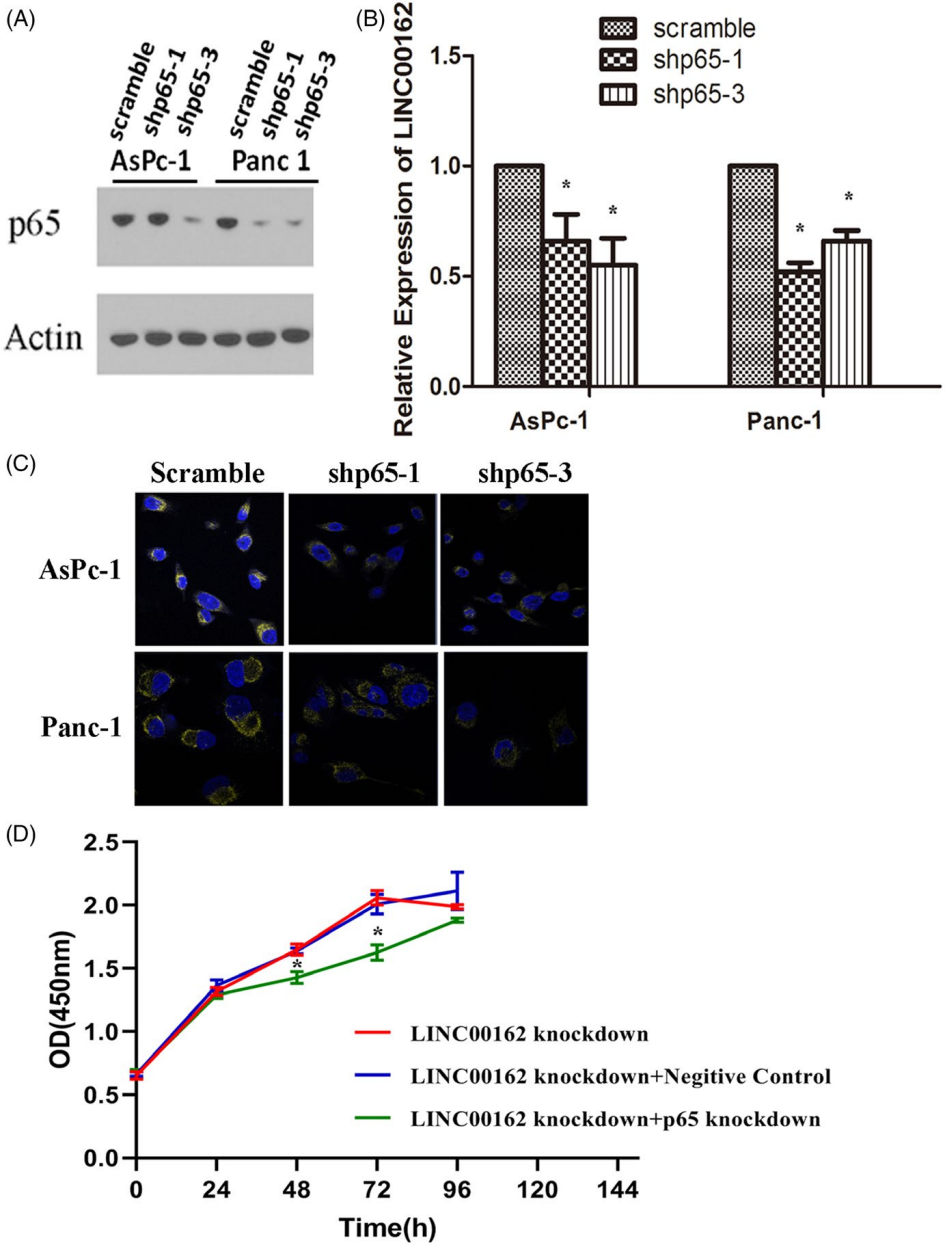
Expression of LINC00162 is regulated by RelA/p65. A‐C, The expression of LINC00162 in PDAC was significantly decreased after RelA/p65 knock‐down. D, The proliferation ability of AsPc‐1 was significantly inhibited after knocking down LINC00162 and RelA/p65 simultaneously

Then, further exploration by knocking down RelA/p65 (Figure 6A) to evaluate the alteration of LINC00162 was also carried out. As shown in Figure 6B‐C, both qRT‐PCR and RNA FISH results evidenced that the expression of LINC00162 in PDAC was significantly decreased after RelA/p65 knock‐down, indicating that the overexpression of LINC00162 was regulated by RelA/p65. A rescue experiment was also performed by knocking down LINC00162 and RelA/p65 simultaneously, and the result of cell proliferation assay indicated that the proliferation ability of AsPc‐1 in LINC00162 knock‐down + RelA/p65 knock‐down group was significantly inhibited, compared to both the LINC00162 knock‐down group and LINC00162 knock‐down + negative control group (Figure 6D). Collectively, these results indicate that RelA/p65 directly involves in the transcriptional regulation of LINC00162.

## 4 DISCUSSION

So far, more than 11 000 lncRNA genes and 18 000 transcripts have been identified^15^ and increasing evidence has indicated that carcinogenesis of different cancer types is closely associated with abnormal expression of lncRNAs. Therefore, lcnRNAs could be proposed as biomarkers or therapeutic targets in cancer.^4^ However, most of their functions remain unknown. The present study investigated the lncRNA expression profiles of PDAC using RNA sequencing technology, and the results identified LINC00162 as the most upregulated lncRNA with the highest mean expression level in PATC cells compared with HPNE. Overexpression of LINC00162 was observed in a series of PATC and PDAC cells using qRT‐PCR and RNA‐FISH. Moreover, knock‐down of LINC00162 suppressed cell proliferation, impaired colony formation ability, promoted cell migration in vitro and inhibited the growth of PDAC xenografts in vivo. The gain‐of‐function study utilizing overexpressed LINC00162 provided similar results. These findings were consistent with previous reports showing that LINC00162 was overexpressed in breast cancer cell lines MDA‐MB‐231,^16^ as well as a study by Piipponen et al,^17^ who observed that LINC00162 promotes tumorigenesis in cutaneous squamous cell carcinoma, suggesting that LINC00162 has an oncogenic role in PDAC and may further act as a novel biomarker or therapeutic target.

However, aside from the study by Piipponen et al,^17^ there is little information available on the function of LINC00162 in cancer, and, based on their observations, LINC00162 was also called PICSAR—namely, p38 inhibited cutaneous squamous cell carcinoma associated lncRNA. The gene coding for LINC00162 is in 21q22.3, located between the protein‐coding genes adenosine deaminase RNA specific B1 (ADARB1) and the family with sequence similarity 207, member A (FAM207A). Interestingly, genome‐wide association studies have found that some alterations in 21q22.3—such as single nucleotide polymorphisms, loss of heterozygosity and copy number alterations—were associated with certain cancers, including prostate cancer,^18^ breast cancer,^19^ salivary gland adenoid cystic carcinoma^20^ and pancreatic cancer.^21^ It remains to be elucidated whether any of these genomic alterations affect the expression or function of the LINC00162 gene.

As for PDAC, KRAS mutation is the well‐known, earliest genetic alteration detected in PDAC development and has been detected in approximately 95% of PDAC cases.^22^ RelA/p65, which is frequently detected in various cancers as a hallmark of cancer development,^23^ is also found constitutively activated in nearly 70% of PDAC tumours and most PDAC cell lines.^2^ Since our lab has previously showed that the NF‐κB pathway is an important downstream pathway of KRAS mutation, and inhibition of NF‐κB activity results in the inhibition of PDAC cell tumorigenesis,^24^, ^25^ this study has sought to confirm if there is any relationship between LINC00162 expression and NF‐κB. As shown in Figure 5, the result of ChIP assay revealed that RelA/p65 directly bound to LINC00162, and further knock‐down of RelA/p65 led to significantly decreased expression of LINC00162, indicating that the expression of LINC00162 was induced by RelA/p65; the proliferation ability of AsPc‐1 also significantly inhibited after knocking down LINC00162 and RelA/p65 simultaneously. Collectively, all these finding indicated that RelA/p65 directly involves in the transcriptional regulation of LINC00162.

In sum, this study has identified LINC00162 as a novel lncRNA, differentially overexpressed in PATC and PDAC cells. In addition, it has found that LINC00162 regulates the proliferation, colony formation and migration of PATC and PDAC cells in vitro, as well as their growth in vivo. These results provide mechanistic evidence that LINC00162 is induced by RelA/p65, and all these findings indicate that LINC00162 is involved in promoting the growth of PDAC and may act as a biomarker and putative therapeutic target in PDAC.

## CONFLICT OF INTEREST

The authors declare that they have no conflict of interest.

## AUTHORS' CONTRIBUTIONS

PC and XQ conceived and designed the study. YL and MW performed most of the experiments. KF and JL conducted the mouse model and histological analysis. JFu and YS processed and analysed the data. YL wrote the manuscript.

## Data Availability

The data that support the findings of this study are available from the corresponding author upon reasonable request.
